# Spatial distribution and traceability of microplastics in the marine atmospheric environment

**DOI:** 10.1016/j.eehl.2026.100287

**Published:** 2026-09-15

**Authors:** Yunlong Li, Shiqi Jiang, Rijian Mo, Ruikun Sun, Zhenqing Dai, Nan Li, Chengyong Li

**Affiliations:** a School of Chemistry and Environment, Guangdong Ocean University, Zhanjiang, 524088, China; b Analytical and Testing Center for Ocean in Western of Guangdong Province, Coastal Ecology Engineering Technology Research Center of Zhanjiang, Analyzing and Testing Center, Guangdong Ocean University, Zhanjiang, 524088, China; c College of Ocean and Meteorology, Guangdong Ocean University, Zhanjiang, 524088, China

**Keywords:** Marine atmospheric microplastic, Atmospheric transport, Source apportionment, Numerical modeling, Physicochemical characteristics

## Abstract

Microplastics (MPs) are emerging atmospheric particulate contaminants of increasing concern due to their global prevalence and capacity for long-range transport. The ocean acts both as a major sink for terrestrially derived MPs and as a secondary atmospheric source, with sea spray aerosols transferring particles from the sea surface through wave breaking and bubble bursting. Across the marine-atmosphere studies reviewed here, reported number concentrations span more than four orders of magnitude (approximately 0.0039–60 items/m^3^), reflecting significant spatiotemporal variability across coastal regions, marginal seas, and remote areas, including the open ocean and polar zones. This review evaluates both active and passive sampling approaches, pretreatment procedures, and analytical methods, emphasizing that the lack of standardized protocols presents a primary obstacle to cross-study comparability. Available observational data are synthesized to describe the global distribution of marine atmospheric MPs, with particular emphasis on suspended concentrations, deposition fluxes, and physicochemical characteristics, including morphology, size, color, and polymer composition. Fibers and low-density polymers, such as polyethylene and polypropylene, are identified as the most aerodynamically mobile fractions. Key factors governing sea–air exchange and atmospheric transport are examined, including wind, precipitation, humidity, and particle-level properties. Various modeling frameworks, including Eulerian, Lagrangian, and observation-driven approaches, are assessed for source apportionment, while the persistent divergence between bottom-up emission estimates and field observations is discussed. Additionally, human exposure pathways and their associated health implications are addressed. Collectively, these findings delineate critical knowledge gaps that currently constrain reliable assessments of the global marine atmospheric plastic budget.

## Highlights

• Elucidated the source-to-sink dynamics where the ocean acts as both a reservoir and a secondary emitter via sea spray aerosols.
• Fibrous microplastics exhibit higher long-range transport potential than fragments due to specific aerodynamic properties and lower settling velocities.
• Critically evaluated the trade-offs between active and passive sampling, emphasizing the urgency of establishing standardized monitoring protocols.
• Identified emission inventory uncertainties and physical parameterization gaps as primary bottlenecks in current Eulerian and Lagrangian traceability models.

## Graphical abstract

**Figure:**
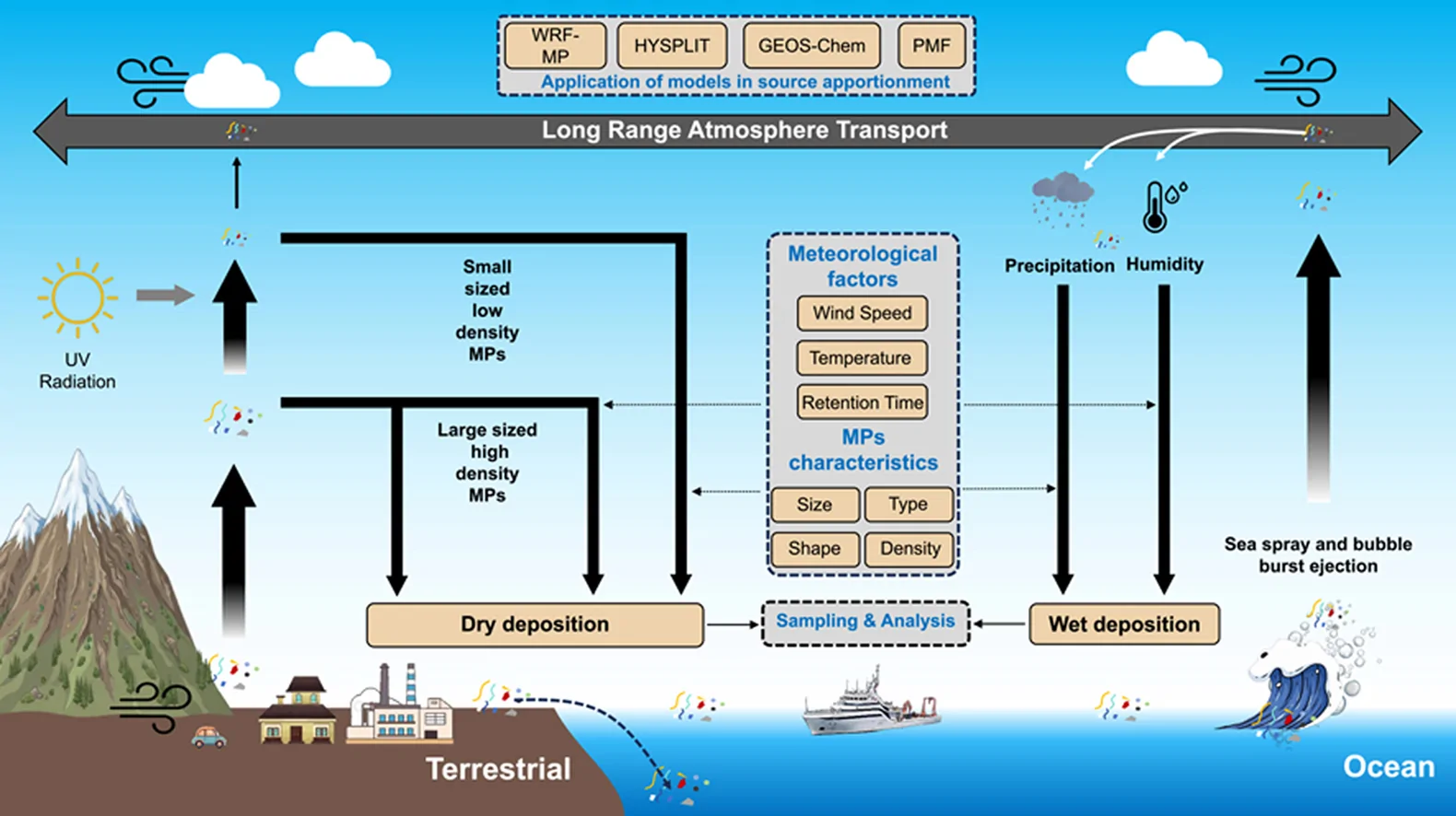

## 1 Introduction

Plastics are synthetic polymeric materials that are widely used owing to their low cost and high durability. In 2024, global plastic production reached 430.9 million tons [1]. However, their inherent resistance to degradation enables prolonged environmental persistence and gradual fragmentation into microplastics (MPs). MPs have been detected across a broad spectrum of environmental compartments, including aquatic systems, soils, and biota [2,3]. Plastic pollution has therefore become a major environmental challenge and a growing threat to planetary health [[4], [5], [6]]. Recent studies have identified the atmosphere as an important pathway for the large-scale transport of MPs [[7], [8], [9], [10], [11], [12]]. Compared with aqueous transport, atmospheric transport is substantially more rapid and operates over far greater distances (Fig. 1). This capacity enables MPs to traverse geographic barriers and reach remote regions, including alpine and polar environments [13,14]. A thorough understanding of the atmospheric fate and behaviour of MPs is therefore essential for assessing global environmental risks and formulating effective mitigation strategies [15,16].

**Fig. 1:**
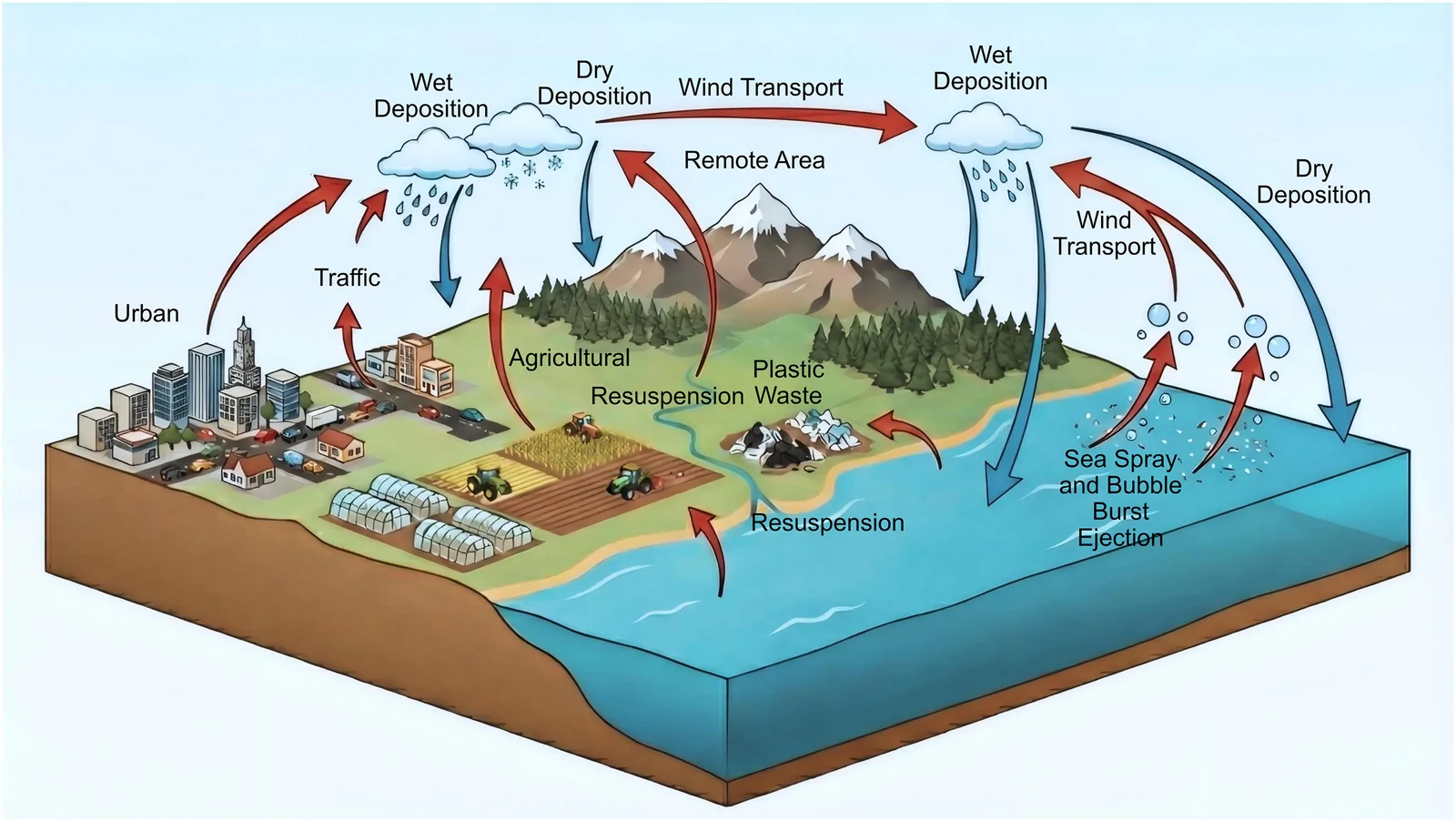
Schematic illustration of the sources, transport pathways, and fate of atmospheric MPs. Red arrows trace the upward emission, resuspension, and subsequent wind-driven transport of MPs. These release pathways originate from terrestrial sources including urban areas, road traffic, agriculture, and open plastic waste, alongside marine emissions driven by sea spray and bubble bursting. Blue arrows indicate the removal of MPs from the atmosphere through wet deposition driven by precipitation and dry deposition driven by gravitational settling. The schematic was generated with the assistance of Google Gemini.

In marine environments, the atmospheric cycling of MPs extends from coastal and marginal seas to the open ocean. This broader marine context warrants attention because sea–air exchange, atmospheric transport, and deposition collectively govern the environmental fate of MPs. Although the ocean has conventionally been regarded as the ultimate sink for terrestrially derived MPs, recent studies have demonstrated that MPs in the sea surface microlayer can enter the atmosphere through the sea–air interface via wave breaking, bubble bursting, and the generation of sea spray aerosols (SSAs) (Fig. 1) [[17], [18], [19]]. Field observations confirm that MPs are present in the atmosphere over both marginal seas and remote oceans [20,21]. Moreover, these data indicate that atmospheric deposition may exceed riverine contributions in certain marine environments [9,22,23].

Although research on atmospheric MPs in terrestrial and nearshore environments has made considerable progress, their spatial distribution and source apportionment across marine environments remain poorly constrained. The difficulty of sampling in the open ocean has confined most studies to coastal or marginal seas [24]. The absence of continuous, global-scale observations means that the spatial distribution of marine atmospheric MPs remains poorly characterized. In the absence of sufficient field data, global estimates must rely heavily on numerical modeling. Nevertheless, current bottom-up calculations and observation-based extrapolations diverge by orders of magnitude [17,19,23,[25], [26], [27], [28], [29]]. This discrepancy arises primarily from the substantial uncertainty in emission inventories and the limited capacity of existing models to represent the distinctive physical attributes of MPs—such as irregular morphology and low density—and their atmospheric aging processes [30,31]. These modelling estimates underscore the challenges of extrapolating to the global scale without high-resolution observations and of adequately parameterizing the factors that govern long-range atmospheric transport [32]. Finally, current methodologies lack robust tracing techniques to distinguish primary emissions from environmentally weathered MPs.

In this review, we examine the source–to–sink dynamics of atmospheric MPs in marine environments and identify the main reasons for the mismatch between model results and field observations. We first assess the limitations of current sampling and analytical methods and highlight the need for standardized protocols. We then synthesize available datasets to describe spatial patterns from coastal waters and marginal seas to the open ocean and remote marine influenced regions. Next, we discuss how meteorological conditions and particle physicochemical properties affect sea–air exchange and long-range transport. Finally, we classify existing modeling approaches and evaluate their applicability under different marine conditions. Overall, the source–sink behaviour of atmospheric MPs in marine environments remains poorly understood, constraining a comprehensive assessment of their interactions across atmospheric, oceanic, and terrestrial systems.

## 2 Literature search strategy

We searched the Web of Science Core Collection on March 14, 2026, for English-language articles published between 2016 and 2026. The search formula TI = (microplastic∗ OR nanoplastic∗ OR microfiber∗ OR plastic particle∗) AND (atmospher∗ OR airborne OR air OR deposition OR transport) was used, and a total of 1005 papers were obtained (Fig. 2). After the removal of one duplicate record, the remaining 1004 records were screened by title and abstract, leading to the exclusion of 851 records. Of the remaining 153 articles, those not directly relevant to marine atmospheric MPs were excluded. After this screening procedure, 34 articles were retained. In total, 38 studies were incorporated into this review, comprising 34 articles retrieved from the primary search and 4 additional studies identified from reference lists (Table S2). The included studies were further categorized by observational setting into coastal, marginal sea, and remote marine/open ocean categories (Table S1). It is important to note that this search strategy primarily targets article titles; therefore, relevant studies indexed solely by their abstracts or keywords are excluded.

**Fig. 2:**
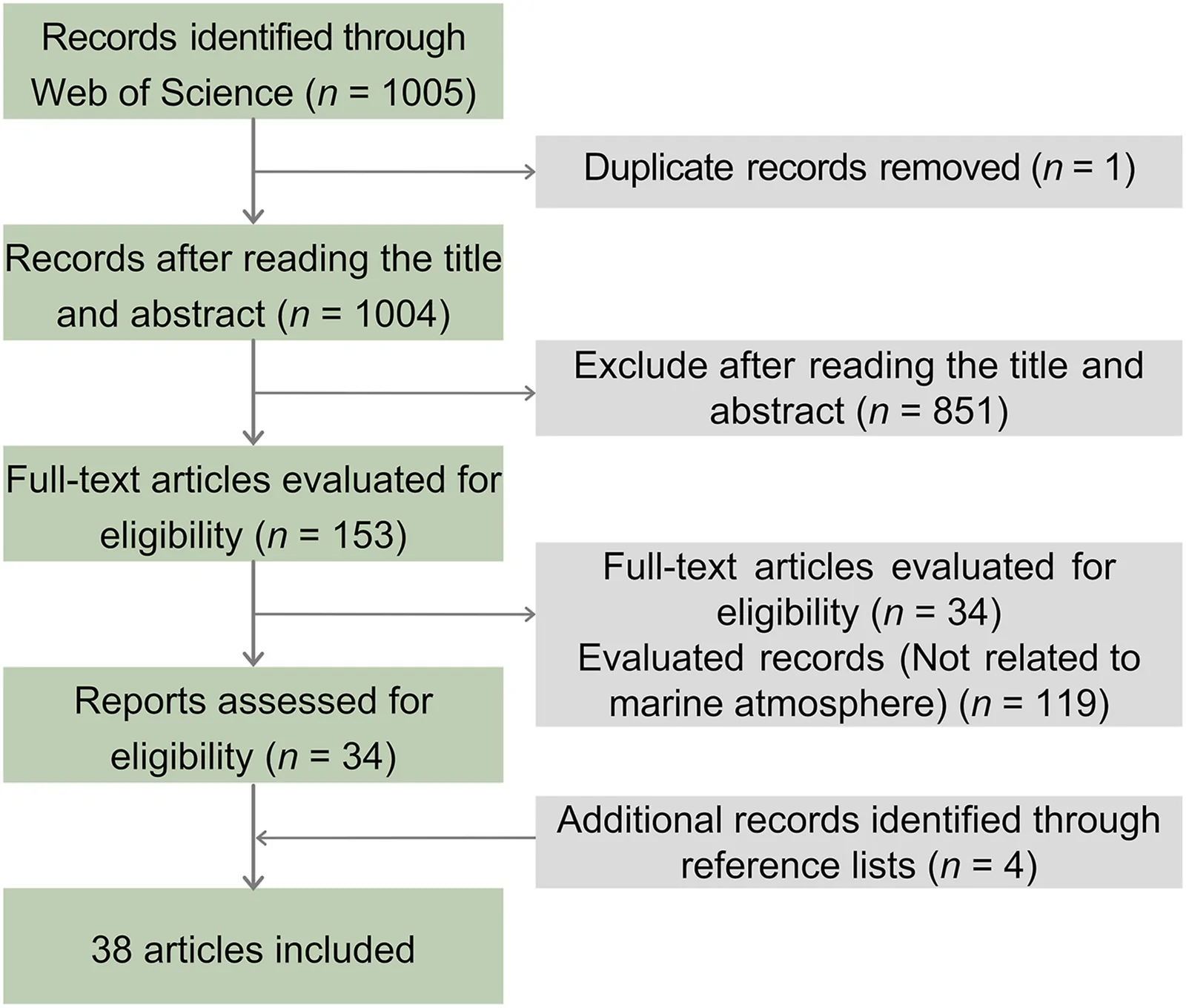
A PRISMA flowchart describing the study selection process. A complete list of the retrieved and screened literature at each stage is provided in Table S2.

## 3 Sampling and analysis of marine atmospheric MPs

### 3.1 MP sampling

The choice of an appropriate sampling approach is a fundamental methodological step in studies of marine atmospheric MPs. Broadly, current approaches can be divided into active and passive sampling. Active sampling measures suspended MPs within a known volume of air and typically reports the results as atmospheric concentrations (e.g., items/m^3^). Passive sampling, by contrast, collects particles that are deposited onto a receiving surface and reports them as deposition fluxes [e.g., items/(m^2^·d)]. The two approaches therefore quantify different components of the atmospheric MP cycle and should not be treated as directly interchangeable. Careful method selection is essential, because it determines whether the resulting dataset reflects the airborne burden available for transport or the cumulative atmospheric input to the ocean surface.

#### 3.1.1 Passive sampling

Passive sampling is primarily used to quantify atmospheric deposition flux, including both dry deposition and precipitation-driven wet deposition. Typical samplers include glass funnels, bulk deposition collectors, wet-only rain samplers, and stainless-steel containers that receive particles settling under gravity or scavenged by rainfall (Fig. 3) [13,33,34]. In the marine atmosphere, this approach is particularly valuable because it directly constrains the atmospheric input of MPs to the ocean surface, thereby providing an empirical basis for regional and global mass balance assessments. This approach is also operationally straightforward. Because passive devices generally require limited power and maintenance, they are well suited to long-term deployments at remote islands, coastal stations, and buoy-based platforms.

**Fig. 3:**
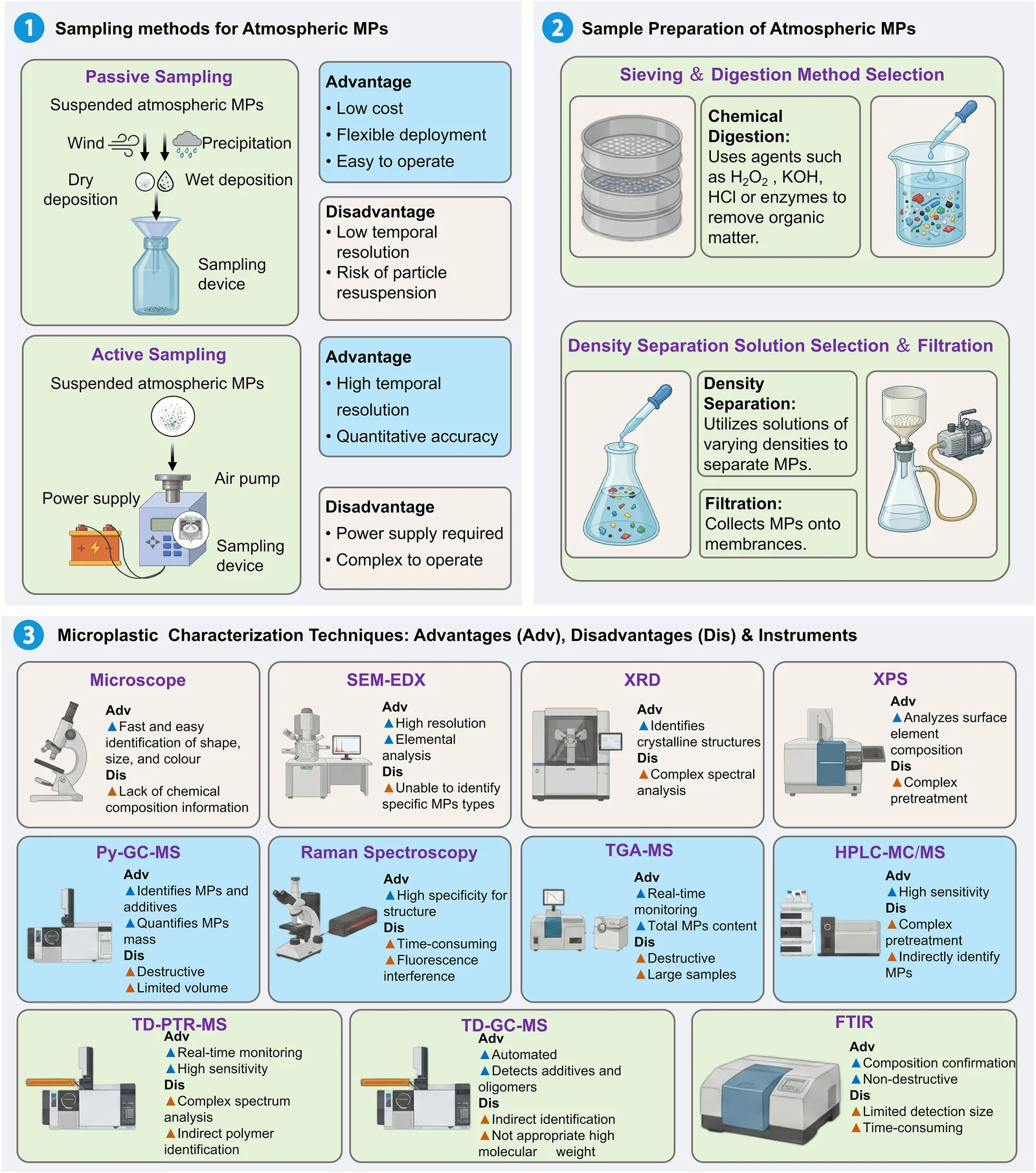
Schematic representation of the analytical workflow for atmospheric MPs. The process includes (1) sample collection via passive sampling (bulk deposition collectors, wet-only samplers) or active sampling (pump–filter systems); (2) laboratory pretreatment, including sieving, chemical digestion (e.g., H_2_O_2_, Fenton’s reagent, NaClO), density separation, and filtration; and (3) instrumental characterization techniques each, with advantages (Adv) and disadvantages (Dis). The icons of instruments were generated with the assistance of Google Gemini.

An additional advantage of passive sampling is its capacity to capture wet deposition, which frequently constitutes the dominant removal pathway for airborne MPs. Where the objective is to distinguish wet deposition from bulk deposition, specialized wet-only collectors can be used to isolate precipitation-associated inputs [35]. This distinction matters because rainfall does not merely dilute the sample; it fundamentally alters the efficiency of particle scavenging from the atmosphere. Passive collectors are therefore especially useful when the goal is to estimate the cumulative load delivered to marine ecosystems over time.

However, passive sampling also has inherent limitations. Collection efficiency may be diminished for small or low-density particles that are readily deflected by airflow or resuspended after initial deposition, potentially leading to systematic underestimation of finer MP fractions. Interpretation of passive sampling data can also be complicated by the temporal variability of precipitation. Because scavenging efficiency depends on rainfall intensity, duration, and event structure, concurrent meteorological observations are often needed to distinguish changes in deposition driven by atmospheric transport from those driven by removal processes [36].

#### 3.1.2 Active sampling

Active pump-based sampling is a well-established technique for atmospheric pollution assessment that has been widely applied over the past decade to quantify diverse atmospheric constituents, including mercury, lead, carbonaceous aerosols, and microorganisms [37]. In recent years, it has also been increasingly used to investigate marine atmospheric MPs. In this approach, a known volume of air is drawn through a filter membrane or stainless-steel mesh over a defined sampling period, allowing suspended particles to be collected for subsequent identification and quantification (Fig. 3). The resulting data are usually expressed as number or mass concentrations per unit air volume.

A major advantage of active sampling is that both sampling duration and processed air volume are explicitly controlled. This improves comparability among sites and sampling periods and enables closer linkage between MP concentrations and concurrent atmospheric conditions. The method can also provide relatively high temporal resolution, ranging from hourly to daily observations, which is useful for resolving short-term variability. In addition, active systems can be adapted to different particle size ranges through the selection of flow rates, inlet configurations, and collection media. For example, Liu et al. [38] installed active samplers at six sites in Shanghai, including measurements at 1.7, 33, and 80 m above ground, using an intake flow rate of 100 ± 0.1 L/min. A comparable protocol was later applied along a transect from Shanghai to the western Pacific Ocean [21].

Despite these advantages, active sampling remains challenging in remote marine environments. High-flow pumps require a reliable power supply, which limits the feasibility of long-term autonomous deployment in open-ocean settings. The method is also sensitive to platform-related interference during shipboard sampling, particularly when inlets are affected by exhaust plumes or deck activities. These issues do not negate the value of active sampling, but they do mean that deployment design and quality control are critical to the reliability of the resulting data.

#### 3.1.3 Sampling strategies

Beyond the choice between active and passive samplers, the sampling strategy dictates which aspect of the atmospheric microplastic cycle is measured. The preceding subsections detail the operating principles and outputs of the two primary types of samplers. However, the overarching sampling design governs the interpretation and comparison of resulting data across studies. This distinction is critical, as suspended particles, dry deposition, and wet deposition represent interrelated yet fundamentally distinct processes. Consequently, they may differ in abundance, size distribution, morphology, and polymer composition.

The sampling strategy should be defined from the outset in accordance with the primary research question. Studies aimed at quantifying near-surface airborne concentrations, identifying transport pathways, or resolving short-term spatial heterogeneity may necessitate a different design compared to studies focused on deposition to the ocean surface or long-term ecological loading. The choice of sampler constitutes only one component of that design. Equally important factors include inlet placement, meteorological context, sampling duration, and contamination control.

In marine environments, the placement of inlets is particularly crucial. The height at which sampling occurs influences the degree to which measurements are associated with freshly generated near-surface marine aerosols, while also impacting the risk of contamination from ship structures, deck operations, and onboard activities. Trainic et al. [20] reported airborne MPs over the remote North Atlantic using a shipboard inlet positioned approximately 15 m above sea level. Their study further demonstrated that materials associated with the vessel and sample-handling procedures could contaminate samples, thereby necessitating stringent procedural blank controls. Consequently, sampling height should be explicitly reported and interpreted alongside vessel layout, wind direction, and the location of exhaust sources. Failing to do so may obscure the boundary between environmental signals and artifacts derived from the platform.

Meteorological conditions should be regarded as integral components of the sampling design rather than merely background information. Studies conducted along the Baltic coast have demonstrated that atmospheric MP deposition is influenced by factors such as wind speed, humidity, precipitation patterns, and air mass trajectories [39]. In the marine atmosphere, wind and wave conditions are also significant, as they affect sea-spray production, which has been proposed as a mechanism for transferring MPs from the ocean surface back into the atmosphere [18,25,28,40]. Recent observations from the northwestern Pacific further suggest that smaller airborne particles dominate in pelagic regions and may better represent signatures of long-range transport [21,41]. These findings underscore the necessity for concurrent meteorological monitoring and, where feasible, backward trajectory analysis. In the absence of such data, variations in concentration or composition may be misinterpreted as spatial patterns, when they may actually reflect changes in transport regimes or scavenging conditions.

The duration of sampling requires strategic adjustments, particularly in remote oceanic settings where airborne microplastic abundance may be low. To obtain robust particle counts, it is essential to sample a sufficient volume of air. However, extending integration times can smooth short-term variability and potentially obscure transient processes. This limitation is particularly significant during extreme weather events. Observations have demonstrated that storms can markedly increase atmospheric microplastic abundance, expand source regions, and intensify wet deposition [42,43]. In such conditions, shorter sampling intervals may be preferable, as they preserve event-scale variability and reduce the risk of conflating pre-storm, storm, and post-storm phases within a single composite sample.

Finally, contamination control is inextricably linked to the sampling strategy in marine studies. Open-ocean measurements are typically conducted at low particle concentrations, making even minor contamination from ropes, paints, textiles, laboratory handling, or ship exhaust capable of distorting the results. Therefore, field blanks, handling blanks, and, where feasible, the screening of onboard materials should be integrated into the sampling design rather than treated as post-corrections. The reliability of marine atmospheric microplastic data relies not only on the sampler itself but also on the overall strategy’s ability to distinguish environmentally transported particles from artifacts introduced by the sampling platform.

### 3.2 Sample preparation

Sample preparation typically encompasses sieving, organic matter digestion, density separation, filtration, and air drying (Fig. 3), each intended to remove interfering materials and enhance analytical accuracy.

As the initial step, sieving is commonly applied during sample pretreatment to remove coarse debris and separate particles into broad size fractions, with the mesh size selected according to the sample matrix and analytical objective. Zhou et al. [44], for example, used 5 mm and 1 mm stainless-steel sieves to fractionate atmospheric deposition samples. Coarse material larger than 5 mm was discarded, the 1–5 mm fraction was examined directly under a stereomicroscope, and particles smaller than 1 mm were collected on a 5 μm membrane filter for further analysis.

Impurities, including those existing independently and attached to MPs, can be removed through digestion using acids [[45], [46], [47]], alkalis [48], enzymes [49,50], and oxidizers [51]. Among these, H_2_O_2_, Fenton’s reagent (H_2_O_2_/Fe^2+^), and NaClO are the most commonly used reagents for digesting atmospheric MP samples [52]. Although NaClO preserves fiber integrity and is suitable for samples with low organic content [[53], [54], [55], [56]], it is less effective than peroxide-based reagents at digesting biological tissues. Conversely, H_2_O_2_ is highly effective at dissolving organic matter and biogenic fibers [57]. However, its digestion efficiency is strongly temperature-dependent [58]. At ambient temperatures (20–25 °C), 30% H_2_O_2_ typically requires 7–8 days to degrade organic impurities [[59], [60], [61], [62]]. Although elevating the temperature to 60–70 °C can shorten the digestion period to less than 24 h [13,44,52], temperatures exceeding 70 °C risk compromising the surface integrity of the recovered MPs [58]. To balance processing efficiency against polymer preservation, Fenton’s reagent (H_2_O_2_/Fe^2+^) is widely recommended. This approach substantially enhances the rate of organic matter decomposition and is increasingly regarded as a promising technique for future MP studies.

Density separation is commonly used to separate MPs from denser inorganic particles based on differences in buoyancy. Following separation, the MP-containing fraction is typically recovered by filtration for subsequent identification and analysis. The efficiency of this procedure depends strongly on the density of the flotation medium and the polymer types present in the sample. In addition, sequential density separation using media of increasing density has also been explored to improve MP recovery while reducing the consumption of high-density reagents [63]. Density separation media commonly applied in MP analyses include sodium chloride (NaCl; about 1.2 g/cm^3^) [64], calcium chloride (CaCl_2_; about 1.35 g/cm^3^) [65], zinc chloride (ZnCl_2_; about 1.78 g/cm^3^) [66], and sodium iodide (NaI; about 1.6 g/cm^3^) [46].

MPs can be concentrated via filtration through membranes. Glass microfiber filters are most frequently used owing to their high efficiency in collecting fine atmospheric particles. When aerosols collected on glass microfiber filters contain relatively few impurities, density separation and digestion steps may be omitted to minimize particle losses [35,38]. Following digestion and density separation, it is recommended that the prepared sample be concentrated on gridded membrane filters with smooth surfaces (e.g., mixed cellulose ester membranes) to facilitate visual observation. The selection of the membrane aperture should consider the minimum size limit of the identification instrument. During sample drying and storage prior to MP identification, oven temperature should be carefully controlled to prevent thermal damage to MP surfaces, including melting or deformation; a temperature not exceeding 70 °C is recommended.

### 3.3 Analysis of MPs

Analytical approaches applied to marine atmospheric MPs are largely consistent with established methodologies employed across environmental research disciplines, typically following a stepwise framework that progresses from physical screening to chemical confirmation.

Visual inspection and optical microscopy constitute the initial stages of physical characterization, enabling classification of MPs by morphological attributes—shape, size, and colour—as well as determination of particle abundance [47,52,67,68]. Surface morphological features are examined microscopically to distinguish synthetic particles from natural fibers. However, the reliability of optical microscopy is constrained by resolution limits; accurate identification of MPs becomes progressively more difficult as particle size decreases below approximately 500 μm. To overcome these limitations and characterize the fine surface textures of smaller MPs, scanning electron microscopy (SEM) has been widely adopted [69,70]. SEM provides high-resolution imaging, and its coupling with energy-dispersive X–ray spectroscopy (SEM–EDX) enables simultaneous elemental composition analysis, thereby facilitating discrimination of MPs from inorganic particles [59]. This coupled approach allows for a detailed assessment of surface weathering and elemental signatures (Fig. 3).

Although morphological characteristics are primarily obtained through microscopic examination, accurate determination of polymer composition relies on spectroscopic techniques. Fourier-transform infrared (FTIR) spectroscopy is widely regarded as the benchmark technique for MP identification. Its widespread adoption stems from the availability of comprehensive spectral reference libraries and its proven capability for resolving characteristic functional groups [71]. In addition, Raman spectroscopy is preferred for analyzing smaller particles, particularly those below 20 μm, owing to its higher spatial resolution. However, Raman signals may be obscured by fluorescence interference arising from additives or organic surface coatings. To mitigate this problem, surface-enhanced Raman scattering (SERS) using silver colloids as active substrates has been developed. This technique significantly enhances the signal efficiency for micro- and nanoplastics (ranging from 100 nm to 10 μm) in aquatic environments, offering a promising avenue for rapid detection [72]. Additionally, fluorescence suppression algorithms have been proposed to further optimize spectral quality [73].

As analytical capabilities have advanced, thermoanalytical and mass spectrometric techniques have been increasingly adopted for more detailed chemical characterization. Unlike non-destructive spectroscopic methods, techniques such as pyrolysis–gas chromatography–mass spectrometry (Py–GC–MS), thermal desorption–GC–MS (TD–GC–MS) [74], and thermogravimetry coupled to mass spectrometry (TGA–MS) [75] are inherently destructive but offer the notable advantage of enabling mass–based quantification. Specifically, Py–GC–MS enables the simultaneous identification of synthetic polymers and organic additives, whereas TD–GC–MS is particularly effective for detecting volatile organic compounds and released oligomers. Advanced methods including high-performance liquid chromatography coupled to tandem mass spectrometry (HPLC–MS/MS) [76], liquid chromatography-mass spectrometry (LC–MS) [77], and thermal desorption-proton transfer reaction-mass spectrometry (TD–PTR–MS) [78] are also employed to identify specific chemical signatures. A detailed comparison of the advantages and limitations of these techniques is provided in Fig. 3.

## 4 Current knowledge of marine atmospheric MPs

Research on marine atmospheric MPs has expanded rapidly in recent years, encompassing diverse environments from coastal regions to remote polar areas. Current evidence reveals a pronounced spatial gradient, with MP concentrations declining markedly from nearshore environments toward the open ocean. Accordingly, this section synthesizes recent empirical observations from across the world’s oceans, with particular emphasis on suspended MP abundances, atmospheric deposition fluxes, and key physicochemical characteristics. To structure these comparisons, the reviewed studies are grouped into three observational tiers: coastal environments, marginal seas, and open-ocean or remote regions. This grouping aligns with the initial literature screening (Table S1) and naturally reflects the distinct spatial gradient in MP abundance, which diminishes from urbanized coastlines toward remote polar zones. Accordingly, this section narrows its focus from the general atmosphere to the coastal–to–open-ocean marine continuum.

Importantly, differences in the lower particle-size detection limits employed across studies can substantially influence reported microplastic abundances. As shown in Table S1, active sampling typically uses glass fiber filters or other filter media, often with a pore size of 1.6 μm, although some studies adopt even smaller size limits (e.g., 0.6 μm). By contrast, passive sampling devices are usually open boxes or bottles, and most of these studies do not use filters during collection. As a result, the particle size range captured by passive devices varies among studies depending on sampler geometry, deployment height, and whether a post-collection filtration step is applied. Atmospheric MPs generally conform to a power-law size distribution, whereby smaller particles are far more abundant and larger ones comparatively scarce. Consequently, the use of filters with smaller pore sizes may yield higher reported abundances. Direct comparison of abundance data across studies without accounting for these detection limits may therefore yield misleading conclusions. Accordingly, we compiled the filter sizes, filter materials, and particle-size ranges reported for each study in Table S1.

### 4.1 Suspended MP abundance

Across studies reporting atmospheric MP number concentrations, mean values compiled in this review span approximately 0.0039–60 items/m^3^, more than four orders of magnitude [[19], [20], [21],^33^,^35^,^38^,^39^,^41^,^42^,[79], [80], [81], [82], [83], [84], [85], [86], [87], [88], [89], [90], [91]]. Atmospheric MP concentrations in coastal cities are consistently higher than those in the marine atmosphere, although substantial spatial heterogeneity remains. For example, atmospheric MP concentrations have been documented in Xiamen, China (0–0.062 items/m^3^) [84] and at sampling sites in Japan (0.12–0.30 items/m^3^) and New Zealand (0.03–0.12 items/m^3^) [88].

Furthermore, discrepancies among measurements from the same region underscore the inherent difficulty of quantifying atmospheric MPs. For instance, a separate study in Shanghai reported a mean atmospheric MP concentration of only 0.08 items/m^3^ [81], substantially lower than the values documented by Liu et al. [38]. Such discrepancies probably reflect the combined effects of methodological differences and spatiotemporal environmental variability.

Measurements of atmospheric MPs across marine regions have generally yielded low concentrations. Observations reported concentrations of 0.008 ± 0.013 and 0.004 ± 0.006 items/m^3^ in the South China Sea and East Indian Ocean, respectively [35], and 0.0046–0.064 items/m^3^ in the Northwest Pacific Ocean [41]. In the Atlantic Ocean, open-ocean number concentrations of 0.06 items/m^3^ have been recorded in a shipboard campaign [20], whereas substantially higher values of 7.7 ± 6.1 items/m^3^ were reported at a coastal Atlantic site where a cloudwater collector was deployed alongside a standard active pump sampler [19]. Across the atmospheric studies summarized above, MP concentrations over marine regions were generally low, particularly in offshore and open-ocean settings. The higher concentrations reported in some studies may reflect the influence of extreme weather events, such as typhoons [43,92] or sampling in coastal megacities [38,83,93], where atmospheric MP levels are often elevatedFig. 4.

The generally low concentrations observed over marine regions are attributable to two principal factors. First, marine regions have fewer direct sources of atmospheric MPs than terrestrial environments and are generally subject to lower levels of human activity. Second, MPs over marine regions are more readily dispersed and diluted by prevailing meteorological conditions and large-scale ocean–atmosphere circulation. This dispersive pattern is reflected in the gradual decrease in MP concentrations from urbanized coastal regions toward the open ocean. For example, Wang et al. [89] reported continuous observations of atmospheric MPs over the open western Pacific Ocean, illustrating this spatial transition. That study reported an average concentration of 0.00841 ± 0.00698 items/m^3^ in the western Pacific. It further showed that MP concentrations decreased markedly with increasing distance from shore and remained far below those reported in coastal urban areas. Chen et al. [86] collected MP samples along a hemispheric cruise route spanning from the central Northern Hemisphere to Antarctica. The samples were predominantly fibers, which were transported more effectively along the transect than fragments. This difference is attributable primarily to differences in particle aerodynamic behaviour. Relative to fragments, fibres typically possess a higher surface area to mass ratio, and consequently experience greater aerodynamic drag that partially offsets gravitational settling. These properties may allow fibers to remain suspended for longer periods and thereby facilitate long-range transport. By contrast, fragment-shaped particles are more likely to settle rapidly, leading to a faster decline in atmospheric concentration with increasing distance from terrestrial sources.

The atmosphere thus acts as a highly dynamic transport medium in which particles are rapidly dispersed and removed, resulting in relatively low yet persistent concentrations. By contrast, the hydrosphere acts as a major reservoir for MP accumulation, continuously receiving inputs from multiple pathways, whereas sediments represent the ultimate long-term sink. Research on MPs in seawater and sediments is far more extensive than that focusing on atmospheric MPs. However, the available datasets still differ markedly in sampling strategy, lower size cutoff, reporting units, and particle-size coverage, which limits direct numerical comparison across environmental media. These relatively low atmospheric concentrations should not be interpreted as evidence that the atmospheric pathway is environmentally insignificant. Instead, they highlight the atmosphere as an efficient transport pathway that can carry MPs from terrestrial sources to remote marine environments, where they may ultimately accumulate.

Because the atmosphere functions chiefly as a transport pathway, concentration measurements alone are insufficient to fully characterize air–sea plastic exchange. A fuller assessment of atmospheric input to marine ecosystems therefore requires attention not only to suspended concentrations, but also to the processes governing particle removal from the atmosphere. This shift requires detailed evaluation of deposition fluxes, which directly quantify the magnitude of MP input to the ocean surface.

### 4.2 Deposition MP flux

Atmospheric deposition constitutes a significant pathway for MP delivery to the marine surface layer [19]. Conversely, MPs present in the marine surface layer can be re-emitted to the atmosphere through sea spray aerosol generation [9,23]. Accurate quantification of atmospheric MP deposition flux is essential for evaluating transport processes at the ocean–atmosphere interface and for constraining the global cycling of MPs. Passive sampling faces multiple technical and operational constraints in open-ocean settings, including elevated contamination risk associated with long-term deployment, pronounced meteorological interference, and insufficient sensitivity for detecting low deposition fluxes. Consequently, the majority of available deposition flux data originate from coastal urban regions, island sites, polar environments, and a limited number of ship-based campaigns [14,85,86]. Although these locations offer relatively stable and manageable sampling conditions, their limited spatial representativeness constrains the ability to characterize global-scale deposition patterns. Beyond these spatial limitations, the currently available deposition flux data are not fully comparable. This inconsistency arises because different components of the deposition process are measured by different studies. To address this issue, bulk deposition, wet deposition, and dry deposition are distinguished in Table S1.

In studies that report wet and dry deposition individually, precipitation is the primary removal pathway for atmospheric MPs. Seasonal monitoring in Shanghai indicated that wet deposition during the rainy months exceeded dry deposition by a factor of approximately 1.5 [93]. Tropical cyclones can amplify this disparity. During and after Typhoon In-Fa, mixed deposition peaked in the week after the typhoon, reflecting storm-driven washout followed by particle resuspension [94]. In other coastal regions, peak deposition fluxes were recorded during typhoon events, declining sharply once storms had passed [92]. Typhoon-driven wet removal also modifies the composition of deposited particles. During Typhoon Sinlaku in the South China Sea, rainfall samples contained more polymer types, more colors, and significantly larger particles than concurrent suspended atmospheric samples, indicating that precipitation preferentially scavenges coarser and more diverse MP assemblages from the air column [42]. Such enhancement is not limited to the western Pacific. As Hurricane Larry traversed the North Atlantic garbage patch and made landfall in Newfoundland, MP deposition reached 1.13 × 10^5^ items/(m^2^∙d), with flux positively correlated with both total rainfall and maximum wind speed [43]. Notably, the predominance of wet removal persists even under non-storm conditions. In Ireland, rainfall accounted for approximately 70% of total atmospheric MP deposition [34]. These observations across contrasting meteorological regimes and ocean basins indicate that precipitation intensity is a primary driver of atmospheric MP loading to coastal and marginal sea environments, although the timing and magnitude of peak deposition vary among events and localities.

Even within the bulk-deposition literature, reported fluxes span several orders of magnitude across coastal settings. Flux ranges from 15 to 624 items/(m^2^∙d) have been recorded in Jakarta, Indonesia [95], Jiaozhou Bay, China [96], Dongguan, China [69], Kochi, India [97], and Yantai, China [44], while higher fluxes were observed in Auckland, New Zealand [98] [4885 items/(m^2^∙d)] and Terengganu, Malaysia [99] [5476 items/(m^2^∙d)]. Moreover, the Malaysian measurements surpassed the average rainy season value in Shanghai, China [93] and the typhoon period flux in the South China Sea [42]. This peak value from Hurricane Larry substantially exceeds all other fluxes cited in this section, underscoring the disproportionate role of extreme weather in episodic MP deposition [43].

### 4.3 Physical characterization: Shapes, size, and colors

#### 4.3.1 Shapes

Environmental MPs display a wide diversity of shapes and sizes [100]. Common morphologies include fibers, fragments, films, foams, pellets, and microbeads [64]. The final morphology of a given particle depends on its primary manufactured form, subsequent weathering processes (e.g., surface erosion), and cumulative environmental residence time.

Fibrous particles frequently dominate marine atmospheric samples across both coastal and open-ocean regions. To visualize these morphological patterns, Fig. 4 presents the proportional distribution of MP shapes across the reviewed literature, based on reporting frequency by region (detailed numerical counts are provided in Table S1). This widespread prevalence of fibers can be largely explained by their distinctive aerodynamic and physical characteristics. Their high aspect ratios and low settling velocities enable fibers to remain suspended for prolonged periods, substantially enhancing their potential for long-range atmospheric transport [101,102]. Furthermore, continuous shedding from textiles and fishing gear provides a widespread and persistent emission pathway for these fibers [103]. In contrast, the relative contributions of other particle morphologies (e.g., fragments, films, and foams) display pronounced regional variability.

**Fig. 4:**
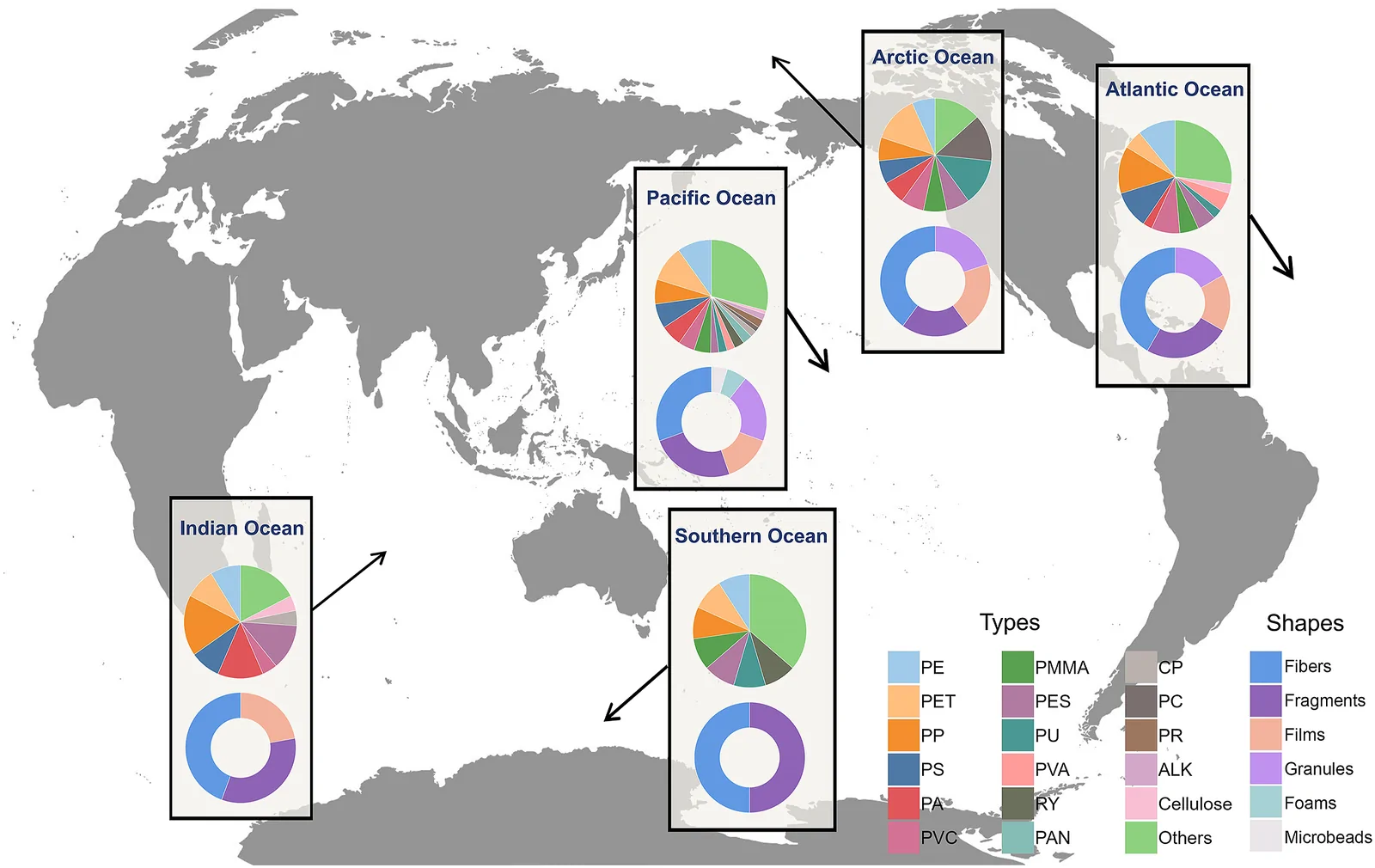
Proportional distribution of MP shapes and polymer types in the atmospheric samples reviewed, organized by major oceanic and continental region. Each pie chart represents the relative reporting frequency of each category, calculated as the number of studies reporting that category as present divided by the total number of studies for that region; underlying numerical counts are provided in Table S1. PS, polystyrene; PE, polyethylene; PP, polypropylene; PET, polyethylene terephthalate; PMMA, polymethyl methacrylate; PES, polyester; PA, polyamide; PR, phenoxy resin; PAN, polyacrylonitrile; CP, cellophane; PU, polyurethane; PVC, polyvinyl chloride; RY, rayon; PVA, polyvinyl alcohol; PC, polycarbonate; ALK, alkyd resin.

Particle morphology directly influences the environmental fate and transport of MPs. For example, planar thin films possess a higher surface area to mass ratio than equivalent fragments, making them more susceptible to wind transport [23,60]. However, the precise role of shape in atmospheric dynamics remains poorly understood and warrants further investigation [9]. Furthermore, specific shapes and size fractions of deposited MPs can cause distinct forms of physical harm to marine biota [104].

#### 4.3.2 Size

Particle size is a dominant factor governing the environmental fate of MPs and their interactions with organisms [105,106]. Because of their irregular morphologies, MPs are typically measured using digital imaging coupled with stereo or compound optical microscopy. While MPs vary widely in size (typically 1 μm to 5 mm) [107], airborne MPs tend to be significantly smaller than those found in aquatic environments and sediments [[108], [109], [110]]. For example, in European and Arctic snow, 80% of detected MPs were ≤25 μm, with 98% measuring <100 μm [14]. Over the South China Sea, non-fibrous particles ranged from 19.45 to 181.08 μm (mean: 77.37 ± 50.79 μm), whereas fibers were considerably longer, ranging from 154.82 to 948.69 μm (mean: 510.12 ± 282.84 μm) [91]. A transect study from the Northern Hemisphere to Antarctica reported average fiber lengths of 1004.80 ± 823.50 μm, while other particle shapes averaged only 166.20 ± 88.60 μm [86]. Similarly, in the atmosphere over Shanghai, the average sizes of fibers, fragments, and other granules were 49.90 μm, 38.40 μm, and 25.00 μm, respectively [83]. These findings consistently indicate that airborne fibers are generally larger than fragments, and that overall MP abundance decreases as particle size increases. Furthermore, particle-size distributions in several marine atmospheric studies fit specific mathematical distributions (e.g., log-normal), highlighting size as a critical parameter for understanding atmospheric transport dynamics [41,69,111].

#### 4.3.3 Colors

Atmospheric MPs exhibit a broad spectrum of colors, including red, orange, yellow, brown, white, gray, blue, and green [14,112].

Specific colors are frequently associated with certain polymer compositions. For example, transparent and translucent particles are often identified as polypropylene (PP), whereas white particles are typically polyethylene (PE) [100]. However, visual identification via stereo microscopy introduces substantial detection bias. Brightly colored plastics are easily recognized against filter backgrounds, whereas dark, white, and transparent particles are often underestimated due to insufficient visual contrast [107,112]. Additionally, particles may experience color fading or alteration due to environmental UV weathering and chemical sample preparation. Consequently, color distributions should be interpreted cautiously and cannot be solely relied upon to infer emission sources. Despite these limitations, reporting color remains ecologically meaningful, as marine organisms often preferentially ingest plastics that visually resemble their natural prey.

In one atmospheric study, blue was the most frequently observed color (36.21%), followed by red (18.97%) and transparent (18.97%) MPs [89]. Another transect study from the Northern Hemisphere to Antarctica revealed morphological differences in color: fibers were predominantly transparent, whereas fragments were primarily green [86]. Overall, blue, transparent, and red are the most frequently detected colors in marine atmospheric MP studies. This prevalence likely stems from a combination of manufacturing preferences, global consumption patterns, and transport mechanisms. For example, transparent polymers are widely used in packaging, mulch films, and shopping bags, whereas blue plastics are frequently associated with maritime and fishing equipment, such as nets and ropes [113]. These general trends imply that the global atmospheric MP cycle is driven by a complex interplay between human consumption habits and environmental transport processes.

### 4.4 Components

Chemical composition represents the most fundamental criterion for the identification and classification of plastic pollution [107]. MPs are chemically diverse and originate from many different product types [112]. The backbone structure of a polymer determines its physical and chemical properties, with plastics broadly classified into thermoplastics and thermosets. Among the polymer types detected, PET (polyethylene terephthalate), PE (polyethylene), PP (polypropylene), PS (polystyrene), and PA (polyamide) are the most frequently reported constituents of marine atmospheric MPs. The available data indicate that MP polymer composition across the five oceans is both broadly consistent and regionally distinct. Common consumer plastic polymers such as PP, PS, and PE, which have a density similar to or lower than that of seawater, account for a high proportion of the MPs detected across all oceans [35,85,86]. This observation corresponds closely with global plastic production patterns, in which PE and PP consistently account for more than half of total output. This strong association suggests that land-based waste sources are likely a major origin of marine MPs. The ubiquitous occurrence of these polymers is further attributable to their intrinsic durability and prolonged degradation times in marine environments [45]. Notably, the Pacific Ocean exhibits the greatest compositional complexity and diversity of atmospheric MPs, with elevated proportions of PA, PS, and PMMA (polymethyl methacrylate), in addition to PE, PP, and PET. In the Atlantic Ocean, PE, PP, and PET remain dominant, whereas other polymer types are reported less frequently. The Indian Ocean is dominated by PP, PA, and PES (polyester). Conversely, the remote ecosystems of the Arctic and Southern Oceans present relatively low MP concentrations. Pollution in these regions is likely to be transported primarily by atmospheric deposition and ocean currents over long distances, rather than by direct local input [114].

Overall, the compositional diversity of marine atmospheric MPs reflects the multiplicity of their sources. When evaluating atmospheric MP pollution, it is essential to consider both land-based atmospheric transport pathways and potential contributions from marine transport processes.

## 5 Factors influencing the transport and deposition of marine atmospheric MPs

The transport and deposition of MPs in the marine atmosphere play a pivotal role in governing their spatial distribution, temporal dynamics, and ultimate environmental fate. This section synthesizes current literature to identify the principal factors controlling the transport and deposition of MPs in the marine atmosphere. Additionally, the underlying mechanisms that govern transport pathways are systematically examined.

### 5.1 Meteorological factors

#### 5.1.1 Wind

Wind is the primary driver of atmospheric MP transport, governing particle dispersion across multiple spatial scales. Turbulence within the atmospheric boundary layer further modulates the vertical mixing and horizontal diffusion of MPs, thereby altering their concentration profiles and deposition rates. Studies have demonstrated that MPs can be transported thousands of kilometers through atmospheric circulation, as evidenced by observational data from remote regions such as the Arctic and the open ocean [9,13]. Allen et al. [13] reported that MPs deposited in the Pyrenees originated from urban areas hundreds of kilometres away, demonstrating the capacity of large-scale atmospheric circulation to transport MPs over regional to continental scales. In marine environments, bubble fragmentation represents a primary mechanism by which MPs are conveyed from the ocean surface into the atmosphere. At the sea–air interface, MPs become embedded within the gel-like matrix of the sea surface microlayer, where they are concentrated prior to aerosol generation. As these aggregates ascend to the ocean surface, intensified wind forcing promotes wave disruption and bubble bursting, facilitating the atmospheric ejection of marine MPs and sustaining a continuous exchange cycle at the air–sea interface (Fig. 5) [18].

**Fig. 5:**
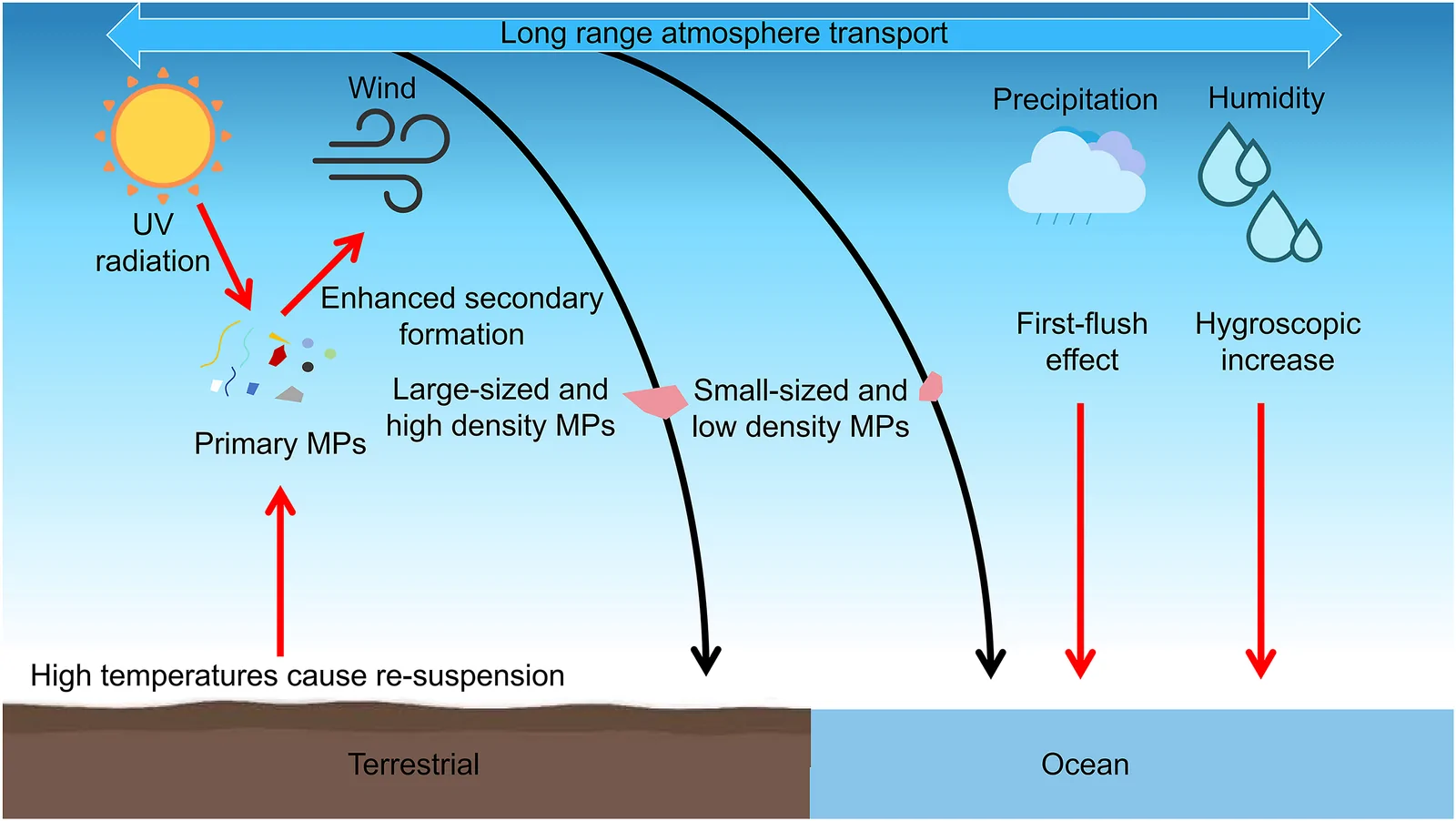
Major meteorological and physicochemical factors governing the transport and deposition of atmospheric MPs. Red labels denote meteorological drivers, including wind, precipitation, temperature, and humidity; black labels denote particle-level properties, including size, shape, density, and surface characteristics. Arrows indicate the direction of influence on transport or deposition processes.

Particle size is a key factor in the aerosolization of MPs by sea spray, although the reported enrichment varies substantially among studies. Yang et al. found extremely strong enrichment for sub-2 μm polystyrene particles under laboratory bubble-bursting conditions in synthetic seawater, with enrichment factor (EF_mixed_) increasing from about 1700-fold at 0.4 μm to 24,000-fold at 1.6 μm [25]. In contrast, Catarino et al. using a miniMART system, reported lower EFs that declined from 19-fold for 0.5 μm particles to 2-fold for 10 μm particles [115]. This difference likely reflects, at least in part, differences in experimental design and in the way enrichment was defined and calculated, meaning that the values are not directly comparable [25,115]. Nevertheless, both studies support the view that smaller MPs are more readily aerosolized than larger ones. A likely explanation is that MPs can accumulate at the interface of rising bubbles and be expelled in jet drops when bubbles burst; because jet drops occupy a limited size range, smaller particles are more easily entrained, whereas larger particles are less efficiently transferred to the atmosphere [25].

#### 5.1.2 Precipitation

Precipitation drives the wet removal of airborne MPs, with deposition fluxes modulated by rainfall intensity and frequency (Fig. 5). Dris et al. [36] demonstrated that rainfall events substantially increase MP deposition in urban and coastal regions, with peak fluxes associated with heavy precipitation. Notably, Yuan et al. [87] reported that deposition fluxes increase exponentially once rainfall intensity exceeds 12 mm/h. In the remote French Pyrenees, Allen et al. [13] reported that MP deposition fluxes are positively correlated with rainfall intensity and duration, but negatively correlated with rainfall persistence. These findings suggest that MP deposition is governed predominantly by the frequency and intensity of precipitation events rather than by prolonged atmospheric residence times. Accordingly, in coastal cities characterized by high rainfall, wet deposition is more efficient than dry deposition at removing airborne MPs [93]. Most atmospheric MPs are removed during the early phases of rainfall, leading to a rapid reduction in atmospheric concentrations [[116], [117], [118]]. This phenomenon, termed the “first-flush effect” [119], demonstrates that MP concentrations in rainwater are highly responsive to both the duration and intensity of precipitation events.

Regional climate patterns markedly influence the significance of wet deposition. In Ireland’s temperate maritime climate, wet deposition accounts for approximately 70% of total atmospheric MP deposition [34]. Brahney et al. [11] reported that snow deposition in remote western U.S. national parks contained substantial levels of MPs. In contrast, wet deposition accounted for only 5% of total atmospheric MP deposition in the arid Shiraz region of Iran [116]. This pronounced contrast underscores the importance of precipitation type and seasonal variability. Ultimately, the combined influence of wet and dry deposition governs the total atmospheric deposition flux [10].

#### 5.1.3 Temperature and humidity

Elevated relative humidity is a defining characteristic of the marine boundary layer and plays a central role in controlling the fate of airborne MPs. Owing to the strong hygroscopic nature of sea salt aerosols, interactions between these aerosols and MPs are substantially intensified. Two dominant physical mechanisms have been proposed: MPs may act as condensation nuclei for water vapor or merge with deliquescent salt particles. Such aggregation promotes hygroscopic growth, thereby increasing particle size and mass. As a result, removal rates through dry gravitational settling and wet deposition are significantly enhanced [21]. This mechanism contributes substantially to atmospheric scavenging processes over the ocean (Fig. 5). The efficiency of this removal pathway explains the consistently lower MP concentrations observed in open seas than in coastal environments [120]. Furthermore, high humidity has been shown to retard photooxidative fragmentation during transport [30]. This preservation favors the deposition of larger particles rather than the formation of secondary MPs. Under saturated conditions, such as ocean fog, MPs are effectively captured through intracloud processes, leading to significant local deposition fluxes [121].

### 5.2 MP characteristics

The physicochemical properties of MPs significantly influence their atmospheric behavior, including their migration efficiency and settling velocity. Particle size is a primary determinant. Smaller MPs (e.g., <10 μm) exhibit longer atmospheric residence times due to reduced gravitational settling and increased susceptibility to turbulent dispersion. Microscale fibrous and fragmented MPs can remain suspended in the air for extended periods, facilitating transboundary transport [86]. In contrast, larger MPs (>100 μm) tend to settle rapidly near pollution sources through dry deposition (Fig. 5) [122].

The shape and density of MPs also exert critical influences. Compared with spherical particles, fibrous MPs, which are frequently observed in atmospheric samples, possess high aspect ratios that decrease settling velocities and promote wind-driven dispersion. Polymer density further governs particle settling behavior in the atmosphere. Low-density polymers such as polyethylene and polypropylene are more prone to long-range atmospheric transport, whereas high-density polymers, including polyethylene terephthalate, tend to deposit closer to their emission sources. Furthermore, surface chemical properties and weathering processes (such as photooxidation) can alter the hydrophobicity and charge characteristics of MPs, thereby affecting their tendency to aggregate with other aerosols and their subsequent deposition behavior [38]. Research indicates that MPs subjected to aging may become more hydrophilic, thereby increasing their adsorption capacity for wet deposition.

### 5.3 Environmental and geographic factors

Environmental and geographic conditions exert a strong influence on the transport and deposition of marine atmospheric MPs. In particular, the combined effects of these factors, meteorological conditions, and particle-specific properties control the spatial distribution and ultimate environmental fate of MPs [13,123]. Coastal areas represent key transition zones where land-based MPs intersect with ocean-derived MPs. Heavily urbanized coastlines exhibit elevated MP deposition as a result of intense local anthropogenic activities, whereas open-ocean regions typically display substantially lower concentrations [124]. This creates a clear pattern of greater pollution near shorelines. Physical geography also plays an important role. Mountain ranges can block air movement, causing MPs to accumulate at high altitudes. This explains why MPs are found even in remote mountain regions [11]. The ocean contributes to atmospheric MP pollution through wave action and bubble bursting. The amount of MPs that enter the atmosphere from the ocean varies by location and sea conditions [20]. Finally, vertical mixing is regulated by the height of the marine atmospheric boundary layer (MABL). Under shallow MABL conditions, the volume available for dispersion is constrained, resulting in the confinement of MPs near the sea surface and a subsequent increase in local concentrations. In contrast, vertical dispersion is enhanced by a deepened MABL, effectively diluting the atmospheric MP load.

## 6 Traceability models of marine atmospheric MPs

### 6.1 Principles and classification of models

Accumulating evidence indicates that atmospheric transport constitutes a key pathway within the global plastic cycle [8]. Numerical modeling approaches are essential for elucidating this process and are predominantly based on Eulerian and Lagrangian frameworks [10]. By representing MP motion from complementary perspectives, these frameworks offer distinct advantages for simulating source attribution, transport dynamics, and deposition processes [9].

Eulerian models calculate the temporal variation in MP mass or number concentration within fixed grid cells. By solving the mass conservation equation, this approach effectively quantifies large-scale source contributions [26]. Mathematically, this approach is founded on the atmospheric advection-diffusion equation. This equation incorporates key physical processes, including wind advection, turbulent diffusion, source emissions, and removal via dry and wet deposition [23].

With respect to source attribution, the Eulerian framework demonstrates strong capability in simulating spatiotemporal concentration fields across entire modeling domains. It can quantitatively estimate contributions from specific source regions or categories (e.g., urban, agricultural, and marine sources) to a given receptor location [125]. Such estimates are commonly obtained through sensitivity analyses of emission inventories or adjoint modeling techniques. Nevertheless, accurate parameterization of the underlying physical processes remains essential. For example, the dry deposition velocity (*V*_d_) depends on the particle size, density, shape, and surface characteristics, and is often estimated via resistance models [101]. Similarly, wet deposition requires parameterizing the scavenging efficiency, which depends on the precipitation type and intensity and on MP physicochemical properties [85]. Additionally, atmospheric aging processes, such as surface oxidation, may alter deposition behavior and must be considered [30].

In contrast, the Lagrangian framework, exemplified by models such as FLEXPART (FLEXible PARTicle dispersion model), traces the trajectories of numerous particles or air parcels driven by wind fields and turbulence [13]. This approach is particularly effective for simulating long-range transport. Within this framework, each particle carries specific data regarding its mass, position, and altitude. Lagrangian particle dispersion models (LPDMs) then use particle trajectories to statistically derive concentration fields and deposition fluxes [126].

Backward trajectory models [e.g., FLEXPART and HYSPLIT (Hybrid Single-Particle Lagrangian Integrated Trajectory model) operated in reverse mode] are widely applied to identify MP sources [91]. Initialized at a defined receptor location, these models reconstruct air-mass pathways over a retrospective time window, typically spanning several hours to multiple days. Subsequently, potential source regions are delineated through trajectory clustering techniques or statistical approaches, such as the Potential Source Contribution Function (PSCF) [94,127]. A critical component of this framework is the real-time computation of dry and wet deposition along individual trajectories. This framework is advantageous for capturing small-scale dispersion near sources or within complex terrain [94]. However, model accuracy relies heavily on the precision of input meteorological fields. Furthermore, the backtracking duration must be set appropriately to cover the typical atmospheric lifetime of MPs [27,43].

A major limitation common to both modeling frameworks is the uncertainty associated with the physical parameterization of MPs. MPs span wide size ranges and exhibit heterogeneous morphologies, variable densities, and electrostatic properties, rendering their dynamic behaviour—including settling velocities and wet scavenging rates—substantially more complex than that of idealized spherical aerosols [102]. As a result, existing models frequently borrow parameterizations from other aerosol classes or depend on scarce empirical observations [128]. Model development must also incorporate the dynamic influences of MP aging, including aggregation and fragmentation, on deposition processes [30].

### 6.2 Application of mainstream models in marine atmospheric MP source apportionment

Building on Eulerian and Lagrangian frameworks, various numerical models have been developed to study the atmospheric lifecycle of marine MPs. By incorporating key physical process parameterizations, these models systematically analyze emission sources, transport pathways, and deposition fluxes. While their temporal scopes, computational efficiencies, and data requirements may differ, they are fundamental tools for understanding critical processes. In particular, they are vital for quantifying terrestrial inputs, assessing contributions from marine resuspension, and revealing transoceanic transport mechanisms [121].

As shown in Table 1 [9,23,[25], [26], [27],^29^,^125^,^129^,^130^], current literature reveals significant discrepancies in estimating atmospheric MP fluxes across different spatial scales and modeling approaches. For global ocean emissions, top-down methodologies yield estimates as high as 8.6 Tg/yr for 0–100 μm [23], whereas a process-based study found only 0.1 Tg/yr from the ocean [29]. Others reported even lower ocean emissions: 1.231 × 10^−3^ Tg/yr for MPs with Dp < 60 μm [22] and 7.73 × 10^−4^ Tg/yr for 0.3–70 μm [25].

**Table 1:** Comparison of atmospheric microplastic flux results from observational data extrapolation and model simulation.

Region	Underlying Model Framework	Estimated flux	Source	Refs.
Global	FLEXPART	6.1 × 10^17^, 2.6 × 10^16^ particles/yr	Terrestrial / marine	[129]
Global	MOZART-4	10, 8 × 10^−4^ Tg/yr	Continental/marine	[130]
Global	CESM	8.6 Tg/yr (oceanic emission)	Ocean/roads/agriculture	[23]
Global	FLEXPART	9.6 Tg/yr (global release), 8.9 Tg/yr (oceanic sources)	Ocean/agriculture/road dust/mineral dust	[27]
Global	GEOS-Chem	0.154, 0.171 Tg/yr	Terrestrial/marine	[26]
Global	Process-based Mechanistic (Bubble Bursting/Jet Drops)	0.1 Tg/yr	Oceanic resuspension	[29]
Global	Experimental Bottom-up (Sea Spray)	7.73 × 10^−4^ Tg/yr	Oceanic resuspension	[25]
Global	FLEXPART	0.1 Tg/yr (TWPs), 0.04 Tg/yr (BWPs)	Road traffic	[9]

Some of the data in the estimated flux are represented by two values, indicating terrestrial emissions and marine emissions. FLEXPART, FLEXible PARTicle dispersion model; MOZART-4, Model for Ozone and Related chemical Tracers, version 4; CESM, Community Earth System Model; GEOS-Chem, Goddard Earth Observing System with Chemistry; TWPs, tire wear particles; BWPs, brake wear particles.

Furthermore, differences in reporting units and particle-size ranges complicate direct comparison among published estimates. Evangelou et al. [129] reduced part of this inconsistency by comparing a compiled global observational dataset with model results over comparable particle-size ranges. Their analysis revealed that conventional models could overestimate global median atmospheric MP concentrations by two to four orders of magnitude, indicating that inferred emission fluxes remain highly sensitive to particle-size assumptions and reporting metrics. Together, these comparisons highlight the extent to which current estimates of marine atmospheric MP emissions remain poorly constrained.

#### 6.2.1 Global chemical transport models

Global-scale chemical transport models have been increasingly applied to simulate the atmospheric transport and deposition of MPs. Brahney et al. [23] implemented atmospheric MPs within the CESM (Community Earth System Model) framework, whereas Fu et al. [26] subsequently developed the GEOS-Chem-Microplastic (GC-MP) model based on GEOS-Chem (Goddard Earth Observing System with Chemistry). Such models are used to quantify the relative contributions of different source regions or source categories to atmospheric MP concentrations and deposition in oceanic and remote environments. These gridded modeling frameworks combine prescribed or optimized emission fields with meteorological transport and deposition processes, allowing the large-scale atmospheric cycling of MPs to be evaluated.

Brahney et al. [23] used global simulations and identified tire abrasion as a dominant source of atmospheric MPs in North America, indicating that the role of marine resuspension had previously been overestimated. Subsequent studies have confirmed that long-range terrestrial transport represents the primary pathway through which MPs are delivered to remote regions, including high-altitude mountain environments and polar areas [8].

However, the source-tracing capability of these models hinges on the accuracy of emission inventories and the parameterization of removal processes [26]. Zhang et al. [10] reported that uncertainties in key inputs, specifically marine resuspension fluxes, cause significant discrepancies (2- to 5-fold) in global flux estimates across different models. Consequently, the absence of unified, high-quality emission inventories remains a primary constraint [9].

#### 6.2.2 Regional meteorology-MPs coupled models

In coastal zones dominated by local circulations (e.g., sea breezes), high-resolution regional coupled models [e.g., Weather research and Forecasting-based microplastics model (WRF-MP)] demonstrate the utility of the Eulerian framework for fine-scale simulations [125]. By incorporating detailed meteorological modules, these models accurately characterize 3D wind fields and turbulent diffusion. This capability is essential for capturing transport driven by complex terrain and local weather systems. While effective for quantifying land‒sea fluxes, these models are constrained by high computational costs and the challenges associated with constructing high-resolution emission inventories [85].

#### 6.2.3 Lagrangian particle dispersion models

Lagrangian modeling approaches, such as FLEXPART and HYSPLIT, are widely applied because of their effectiveness in identifying source regions via air-mass trajectory analysis [13]. These models simulate the movement of large numbers of particles either forward in time (to estimate concentrations and spatial distributions) or backward in time (to infer potential source regions) from a defined receptor location [84,123,131].

Using backward FLEXPART simulations, Allen et al. [13] demonstrated that MPs in the remote Pyrenees originated from urban areas over 100 km away. This study illustrates the utility of Lagrangian trajectory models for identifying potential source regions and assessing regional transport pathways of atmospheric MPs. However, these models are sensitive to the quality of meteorological inputs, while uncertainties in particle-settling behavior and deposition processes may further affect the reliability of inferred source regions [132].

#### 6.2.4 Observation-driven and empirical models

Observation-driven and empirical approaches, such as positive matrix factorization (PMF), rely on observational datasets and use polymer composition, particle morphology, and related characteristics to infer potential source contributions. These approaches can provide useful complementary evidence for source apportionment without directly depending on emission inventories or physical transport simulations [34,94].

However, their application to atmospheric MPs remains constrained by the limited availability and representativeness of source-related compositional signatures, methodological heterogeneity among studies, and the potential overlap of characteristics among different source categories. Accordingly, receptor-based analyses are better viewed as supportive rather than definitive tools for source attribution. For example, Bergmann et al. [14] combined μ-FTIR with PMF in Arctic snow samples and identified ship-derived paint particles and tire wear debris as key contributors. Such compositional evidence is informative for relative source interpretation, but it cannot by itself resolve precise geographic source regions or transport pathways [121].

#### 6.2.5 Model selection and source tracing capability comparison

To make the comparison of modeling approaches more useful for interpretation and study design, the main model classes discussed in this review are summarized in a decision matrix (Table 2). Rather than comparing models only in terms of generalized strengths and limitations, the matrix links each class to its main application, minimum required inputs, recommended validation, and outputs that remain poorly constrained in current studies of marine atmospheric MPs.

**Table 2:** Decision matrix for the selection of source apportionment models for atmospheric MPs.

Model	Main application	Minimum required inputs	Recommended validation	Outputs
Global chemical transport models	Large-scale source contributions, long-term source–sink balance	Emission inventories, meteorological data, deposition parameterization	Measured atmospheric concentrations and deposition fluxes, large-scale land–ocean and coastal-remote gradients	Oceanic emission fluxes, global source apportionment, effects of size distribution on estimated emissions
Regional meteorology coupled models	Regional transport, land–sea exchange	Regional meteorological data, MPs properties, dry/wet deposition schemes	Site observations, regional deposition fluxes, changing meteorological conditions	Regional source apportionment, deposition of non-spherical particles
Lagrangian particle dispersion models	Transport pathways, potential source regions	Receptor or release location, meteorological data, transport duration	Sensitivity to trajectory height and transport time, consistency with observed events	Potential source regions and transport pathways; quantitative source contributions when coupled with dispersion/inverse methods
Observation-driven and empirical models	Source contribution analysis, regional transport	MPs type or composition, hourly concentration data	Clustering results, consistency with source characteristics	Relative source contributions and source profiles; limited geographic source and transport-pathway resolution

Global-scale models, such as GC-MP, are commonly employed to evaluate the relative contributions of diverse continental and oceanic regions. Nevertheless, the reliability of these simulations is limited by uncertainties in existing global emission inventories [133]. At regional scales, MP transport processes can be represented with higher fidelity using high-resolution regional models, such as WRF-MP, particularly within coastal environments influenced by complex land–sea breeze systems. Despite these advantages, the practical application of such models is restricted by substantial computational requirements and the need for highly resolved emission inventories [125].

Lagrangian approaches such as FLEXPART efficiently identify pollution sources influencing specific receptor locations [84,131]. Their intuitive trajectory-based outputs provide clear insight into atmospheric transport pathways. Nonetheless, uncertainties may arise from the simplified representation of deposition and removal processes. For studies that require the apportionment of contributions from distinct source categories, including tire wear particles, textile fibers, and ship-derived paints, receptor models, such as PMF, represent the most direct analytical approach. However, the effectiveness of this method is strongly dependent on the sampling strategy and the representativeness of the collected samples.

Notably, complex source apportionment challenges are often inadequately addressed by single models employed in isolation. Consequently, the development of multi-model coupling frameworks represents a priority for future research. For example, the path analysis capability of Lagrangian models can be integrated with the source resolution functionality of receptor models. Alternatively, boundary conditions for regional simulations can be provided by global models. Concurrently, fundamental research on the physicochemical behavior of MPs must be advanced. Consequently, parameterization schemes across various models need to be refined. In particular, further investigation of key processes, such as morphological evolution during atmospheric aging and hygroscopic growth, is needed.

Moreover, the limitations inherent to conventional modeling frameworks are increasingly mitigated through the application of emerging machine learning (ML) approaches. In particular, ML techniques are utilized to reduce uncertainties associated with emission inventories and physical parameterizations. These algorithms are well-suited to capturing complex, nonlinear interactions that characterize MP transport processes. Importantly, the integration of ML methods with physics-based models represents a promising direction for future research. This hybrid modeling strategy improves computational efficiency while maintaining the physical interpretability of model outputs [[134], [135], [136]].

### 6.3 Model emission fluxes and source identification

Comparisons among published model estimates further illustrate how strongly inferred source contributions depend on model framework, particle-size definition, and reporting metric. Early extrapolations suggested global oceanic emissions approaching 8.6 Tg/yr [23]. This represents 98% of the total emission flux from all sources. However, its wide confidence interval of 0–22 Tg/yr reflects the uncertainties in estimating sea–air emissions using the inversion of regional observations distant from oceans. Subsequently, using the observational dataset from Brahney et al. [23] and inverse modeling with FLEXPART, Evangeliou et al. estimated total global atmospheric MP emissions of 9.6 Tg/yr for particles up to 250 μm, of which oceanic emissions accounted for 8.9 Tg/yr (93% of the total) [27]. Furthermore, drawing upon insights from Brahney et al. [23]regarding particle size and residence time, as well as employing the same optimal estimation approach, Fu et al. estimated the global sea–air emission at 0.171 Tg/yr (53% of the total sources), using GEOS-Chem and global observational data [26]. However, as previous studies have indicated, whether oceanic emissions can account for such a large fraction of atmospheric microplastics remains questionable. Collectively, these modeling estimates highlight challenges in scaling globally without the benefit of high-resolution observations, and oversimplifying factors in particle long-range atmospheric transport [32].

Subsequently, Yang et al. estimated the global sea–air flux of sub-70 μm MP at 7.73 × 10^−4^ Tg/yr [25], which is 10^4^ times lower than the previous estimates [23,27]. More recently, Yang et al. [130] adopted the low oceanic emission flux derived from previous bottom-up sea-spray experiments [25] to simulate the global distribution of sub-70 μm atmospheric MPs using MOZART-4 (Model for Ozone and Related chemical Tracers, version 4). Their simulations suggest that, under the adopted low-ocean-emission scenario, the ocean acts predominantly as a sink rather than a source of atmospheric MPs, contributing only about 0.008% of global atmospheric MP emissions while receiving approximately 15% of total atmospheric deposition [130]. Subsequently, Evangelou et al. compiled a global dataset of atmospheric MPs and compared it with model results over comparable particle-size ranges. Their analysis showed that previously published models overestimated global median atmospheric MP concentrations by two to four orders of magnitude. Median concentrations were reported to be 27-fold lower over the ocean than over land, and total global land-based and oceanic emissions were estimated at 6.1 × 10^17^ and 2.6 × 10^16^ particles/yr, respectively. The results indicate that atmospheric MP numbers are dominated by land sources and that emission size distributions remain a major source of uncertainty [129].

Additionally, several major terrestrial sources of atmospheric MPs have been identified by existing models. Road traffic is considered the primary terrestrial pollution source by most global transport models [9,27,136]. Brahney et al. showed that atmospheric MPs in the western United States are derived mainly from secondary re-emission sources. These comprised road dust (84%), oceanic emissions (11%), and agricultural soil dust (5%) [23]. As a supplement to the global perspective, marine air masses are typically tracked to densely populated coastal cities by trajectory analysis. Through this approach, potential source regions and the dominant transport pathways of MP-laden air masses can be reasonably inferred [84,123,131,137,138].

## 7 Human exposure pathways and potential health implications

Understanding how MPs in the marine atmosphere reach human receptors is essential for evaluating their potential health risks. Human exposure to MPs occurs through oral ingestion, inhalation, and dermal contact, with oral ingestion generally recognized as the primary route of exposure [139]. However, in marine atmospheric environments, inhalation poses the most immediate concern, as airborne MPs may be inhaled during coastal and maritime activities. Indirect exposure primarily occurs through the food chain, as MPs ingested by marine organisms can be transferred across trophic levels and ultimately enter the human diet. Additionally, atmospheric deposition of MPs onto the sea surface further increases the concentration of MPs in coastal waters. These particles can be resuspended into the atmosphere or re-enter nearshore waters, creating a continuous cycle between the atmospheric and aquatic environments.

The health significance of inhaled MPs is largely influenced by their size and shape. Particles within the few micrometre range primarily deposit in the upper airways and are subsequently cleared by mucociliary mechanisms. In contrast, sub-micrometre and ultrafine fractions (<100 nm) penetrate more effectively into the bronchiolar and alveolar regions, where their deposition efficiency is markedly increased [139]. Studies have confirmed the presence of MPs larger than 3 μm in human lung tissue [[140], [141], [142]]. However, analyses of bronchoalveolar lavage fluid have failed to detect particles smaller than 5 μm, indicating that current analytical methods may underestimate the abundance of finer fractions, rather than suggesting their absence. For instance, recent estimates suggest that over 13% of airborne PM_2.5_ consists of MPs [143]. In certain urban areas, the concentration of MPs in the air can reach tens of thousands of particles per cubic meter [144]. Recent estimates indicate that adults may be exposed to over 10,000 MPs through the air annually [145], potentially posing significant risks to human health [146]. Fibers warrant particular attention due to their elongated geometry, which prolongs atmospheric suspension and increases the likelihood of inhalation. In contrast, nanoplastics raise concerns regarding their ability to penetrate deeper tissues and potentially translocate into the circulatory system. However, exposure levels are not uniform. Factors such as wind speed, humidity, wave state, and boundary-layer structure influence the abundance of MPs near the surface, introducing additional variability. Fishers, port workers, aquaculture personnel, and ship crews frequently operate in environments where sea spray generation and the mechanical abrasion of fishing gear result in episodic concentration peaks, which routine area-averaged monitoring fails to capture.

MPs should not be regarded as physically or chemically inert. Their surfaces have the capacity to adsorb trace metals and hydrophobic organic contaminants, while the polymers themselves may gradually release additives such as plasticizers, flame retardants, and stabilizers over time [139,147]. Furthermore, microbial colonization introduces an additional layer of concern. The plastisphere, present in both aquatic and terrestrial ecosystems, is generally enriched with potential pathogens, antibiotic resistance genes, and viruses compared to the surrounding environment [148]. In the atmosphere, MPs can transport and sustain microorganisms over long distances due to their low density, irregular shape, and surface properties, which prolong airborne residence and provide protection against UV radiation and desiccation. Recent observations have indicated significant positive correlations between airborne MP concentrations and indicators of microbial pathogenicity in particulate matter samples [148,149]. Although current evidence is insufficient to establish marine atmospheric MPs as a validated infection vector, the potential for simultaneous chemical and microbial co-exposure complicates risk evaluation beyond the capabilities of single stressor frameworks.

Furthermore, these risks are not spatially uniform. Exposure in a densely urbanized estuary differs significantly from that on a remote island or aboard an offshore vessel, and many coastal environments reflect the converging influences of sea spray, shipping, fisheries, tourism, and nearby urban emissions. Very few studies have quantified personal MP exposure in marine or coastal atmospheric environments, and even fewer have linked environmental measurements to biologically meaningful dose metrics, leaving the actual health consequences largely uncharacterized. Currently, no reference threshold values exist for MPs; however, an analogy to established air quality guidelines for PM_2.5_ and PM_10_ has been proposed as a feasible starting point [150], with the reference dose for inhaled MPs estimated from MP concentrations in size-fractionated aerosol samples [139]. Advancing this area requires size-resolved monitoring that captures the entire respirable spectrum, including nanoplastics, targeted studies in coastal and occupational microenvironments, and systematic assessments of chemical and biological co-contaminants. Ultimately, environmental monitoring must be coupled with toxicological and epidemiological evidence to translate exposure data into risk assessments that are applicable to vulnerable coastal and maritime populations.

## 8 Conclusions and future perspectives

This review synthesizes current knowledge of MPs in the marine atmospheric system. Observational evidence consistently demonstrates a spatial gradient in which MP abundance declines from urbanized coastlines toward the open ocean, reflecting the predominance of terrestrial inputs. Fibrous particles and low-density polymers (PE and PP) dominate across all sampled environments—a composition that mirrors global production patterns—and fibrous morphologies exhibit the greatest long-range transport potential owing to their low settling velocities and high surface area to mass ratios. Transport and deposition are jointly governed by meteorological forcing and particle-level properties: wind drives horizontal dispersion and modulates bubble-bursting dynamics at the sea surface; precipitation constitutes the primary removal pathway, with extreme weather events capable of amplifying deposition fluxes by orders of magnitude; and elevated humidity accelerates particle removal through hygroscopic growth of MP-aerosol aggregates. Despite progress in Eulerian, Lagrangian, and observation-driven modelling, bottom-up emission estimates and field observations diverge by several orders of magnitude—the central unresolved challenge in this field—owing to the absence of standardized protocols, incomplete emission inventories, and inadequate parameterization of non-spherical aerodynamics and atmospheric aging processes. Meanwhile, the potential for human inhalation exposure during coastal and maritime activities remains poorly quantified, with no validated reference thresholds yet established.

To advance the field, several priorities merit attention. First, globally harmonized sampling, pretreatment, and identification protocols, together with standardized reporting units and particle size classifications, are prerequisites for cross-study comparability and the assembly of a reliable global database. Second, fundamental research on MP aging, including photooxidation, biofilm formation, and their consequences for settling velocity, hygroscopicity, and surface charge, is essential before transport model fidelity can improve substantially. Third, multimodel coupling frameworks that integrate the trajectory-tracing capability of Lagrangian models with the source-apportionment strengths of Eulerian and receptor approaches, augmented by machine-learning techniques for capturing non-linear transport dynamics, should be developed. Finally, targeted field observations and controlled laboratory experiments are urgently needed to constrain the sea-to-air MP flux; bubble-bursting dynamics and sea surface microlayer behaviour remain among the most poorly quantified components of the global plastic cycle, and closing this gap is indispensable for any credible atmospheric plastic budget assessment.

## CRediT authorship contribution statement

**Yunlong Li:** Writing – original draft, Conceptualization. **Shiqi Jiang:** Writing – review & editing, Data curation. **Rijian Mo:** Methodology, Investigation. **Ruikun Sun:** Resources, Formal analysis. **Zhenqing Dai:** Writing – review & editing, Validation. **Nan Li:** Writing – review & editing. **Chengyong Li:** Writing – review & editing, Supervision, Project administration.

## Declaration of generative AI use

During the preparation of this work, schematic illustrations and cartoons were partially generated with the assistance of Google Gemini. After using this tool, all the authors reviewed and edited the content as needed ensuring accuracy and originality of any AI generated images and take full responsibility for the content of the publication.

## Declaration of competing interest

The authors declare no competing interests.
